# Thyroid Function and Perfluoroalkyl Acids in Children Living Near a Chemical Plant

**DOI:** 10.1289/ehp.1104370

**Published:** 2012-03-27

**Authors:** Maria-Jose Lopez-Espinosa, Debapriya Mondal, Ben Armstrong, Michael S. Bloom, Tony Fletcher

**Affiliations:** 1 Department of Social and Environmental Health Research, London School of Hygiene & Tropical Medicine, London, United Kingdom; 2 Department of Environmental Health Sciences, and; 3 Department of Epidemiology and Biostatistics, School of Public Health, University at Albany, State University of New York, Rensselaer, New York, USA

**Keywords:** children, PFAA, PFNA, PFOA, PFOS, T_4_, thyroid disease, thyroid hormones, TSH

## Abstract

Background: Animal studies suggest that some perfluoroalkyl acids (PFAAs), including perfluorooctanoate (PFOA), perfluorooctane sulfonate (PFOS), and perfluorononanoic acid (PFNA) may impair thyroid function. Epidemiological findings, mostly related to adults, are inconsistent.

Objectives: We investigated whether concentrations of PFAAs were associated with thyroid function among 10,725 children (1–17 years of age) living near a Teflon manufacturing facility in the Mid-Ohio Valley (USA).

Methods: Serum levels of thyroid-stimulating hormone (TSH), total thyroxine (TT_4_), and PFAAs were measured during 2005–2006, and information on diagnosed thyroid disease was collected by questionnaire. Modeled *in utero* PFOA concentrations were based on historical information on PFOA releases, environmental distribution, pharmacokinetic modeling, and residential histories. We performed multivariate regression analyses.

Results: Median concentrations of modeled *in utero* PFOA and measured serum PFOA, PFOS, and PFNA were 12, 29, 20, and 1.5 ng/mL, respectively. The odds ratio for hypothyroidism (*n* = 39) was 1.54 [95% confidence interval (CI): 1.00, 2.37] for an interquartile range (IQR) contrast of 13 to 68 ng/mL in serum PFOA measured in 2005–2006. However, an IQR shift in serum PFOA was not associated with TSH or TT_4_ levels in all children combined. IQR shifts in serum PFOS (15 to 28 ng/mL) and serum PFNA (1.2 to 2.0 ng/mL) were both associated with a 1.1% increase in TT_4_ in children 1–17 years old (95% CIs: 0.6, 1.5 and 0.7, 1.5 respectively).

Conclusions: This is the first large-scale report in children suggesting associations of serum PFOS and PFNA with thyroid hormone levels and of serum PFOA and hypothyroidism.

Thyroid hormones play important roles in regulating metabolism, growth, and development, especially in normal brain maturation and development (Porterfield and Hendrich 1993). They regulate the processes of neurogenesis, denditric and axonal growth, synaptogenesis, and myelination (Bernal 2007). It is now recognized that even slight differences in the concentration of thyroid hormones during pregnancy or after delivery may be associated with neurological impairment (Freire et al. 2010; Pop et al. 1999). Thyroid hormones are also essential for children because some neurodevelopmental processes, such as myelination, are not completed until adolescence (Rice and Barone 2000), and they are also important for the behavior and cognitive function of the young and adolescent brain (Anderson 2001). In addition, thyroid hormone deficiency causes growth delay, precocious puberty in both sexes, and hirsutism in females (Papi et al. 2007).

There is a growing concern that environmental toxicants may be related to thyroid impairment (Boas et al. 2009). Animal studies have suggested that exposure to some perfluoroalkyl acids (PFAAs), including perfluorooctanoate (PFOA, also called C8), perfluorooctane sulfonate (PFOS), and perfluorononanoic acid (PFNA), may interfere with thyroid status (Lau et al. 2007; Liu et al. 2011). There had been some concern that these apparent associations may derive from the analysis of analog free thyroxine (FT_4_) being affected by the presence of PFOS, as observed in rats highly exposed to PFOS (Chang et al. 2007). However, such bias has not been observed in a human population with typical U.S. serum PFOS concentrations but higher PFOA concentrations (Lopez-Espinosa et al. 2011a).

Published epidemiological findings are not consistent (Steenland et al. 2010), have generally focused on adults, and have been cross-sectional in nature—leaving a gap in our understanding of possible PFAA effects in children. Whereas associations were reported for some PFAAs and thyroid hormones in some studies of nonoccupational exposure (Dallaire et al. 2009; Kim et al. 2011; Knox et al. 2011), others found little or no evidence of associations (Bloom et al. 2010; Chan et al. 2011; Emmett et al. 2006). Studies in occupational settings also reported some statistically significant associations with thyroid hormones, although at high PFAA concentrations (Olsen and Zobel 2007; Olsen et al. 2003). A higher odds ratio (OR) of reported thyroid disease was recently associated with PFOA or PFOS exposure in the National Health and Nutrition Examination Survey (NHANES) population (Melzer et al. 2010).

PFOA has been used in the manufacture of fluoropolymers at a chemical plant in Parkersburg, West Virginia, since 1951. In 2001, a group of residents from the Ohio and West Virginia communities in the vicinity of the Washington Works plant filed a class-action lawsuit, alleging health damage due to contamination of human drinking-water supplies with PFOA. The settlement of this lawsuit led to a baseline survey, called the C8 Health Project, conducted during 2005 and 2006 on residents who lived in six contaminated water districts surrounding the chemical plant, as described by Frisbee et al. (2009). In the Mid-Ohio Valley child population 8–18 years of age, PFOA concentrations were markedly higher (Lopez-Espinosa et al. 2011b) than in 2005–2006 NHANES children 12–19 years of age (median = 23 vs. 3.8 ng/mL, respectively) (Kato et al. 2011).

Assessment of possible PFAA effects on thyroid hormone function in children is of special interest for the reasons outlined above. Accordingly, we designed the present study to estimate associations of thyroid function with *a*) modeled *in utero* PFOA concentrations and *b*) measured serum PFOA, PFOS, and PFNA concentrations collected during 2005–2006 in children 1–17 years of age from these Ohio and West Virginia communities.

## Methods

*Study population.* The C8 Health Project enrolled participants between August 2005 and July 2006. All participants gave written informed consent before inclusion: Parents or guardians provided consent on behalf of children. The London School of Hygiene & Tropical Medicine Ethics Committee approved this study. The purpose of the Project was to collect health data from members of the class action lawsuit through questionnaires and blood tests, including measurements of PFAAs. Individuals were eligible to participate in the C8 Health Project if they had consumed water for at least 1 year between 1950 and 2004 from six contaminated water districts or private wells in proximity to a Teflon manufacturing facility. The C8 Health Project collected data on 69,030 people, of whom 12,476 were 1–17 years of age at enrollment. Participation rates for age groups 5–10, 11–14, and 15–19 years, and residing in the area at the time of the survey, were 77%, 87%, and 95%, respectively (Frisbee et al. 2009). Of the 12,476 children, 10,725 (86%) had serum PFAA and thyroid hormone measurements or information on reported thyroid diseases (from questionnaire responses), and were included in the present analyses. Within this population, 4,713 children were successfully matched to their mothers (also participating in the C8 Health Project) (Mondal et al. 2012); effects on child thyroid function in relation to modeled *in utero* PFOA exposure was also estimated for this subsample.

*PFAA determinations.* Laboratory analyses of PFAA were conducted by a commercial laboratory (Exygen, State College, PA, USA). Samples collected at survey were analyzed for 10 PFAAs including PFOA, PFOS, and PFNA. The laboratory analytical methods and quality control procedures have been described elsewhere (Frisbee et al. 2009). Briefly, serum concentrations of PFAA were determined using liquid chromatography separation with detection by tandem mass spectrometry. Estimates of precision for PFOA were within ± 10% for multiple replicates over the range of 0.5–40 ng/mL, with a more precise relative precision measure of approximately 1% for highly fortified (10,000 ng/mL) samples. Relative precision estimates for PFOS and PFNA were similar to those for PFOA. The detection limit (LOD) was 0.5 ng/mL, and observations below the LOD were assigned a value of 0.25 ng/mL (*n* = 0, *n* = 16, and *n* = 107 in the case of PFOA, PFOS, and PFNA, respectively, for this study population).

Historical PFOA exposures for all participants in the C8 Health Project were estimated through environmental, exposure, and pharmacokinetic modeling in conjunction with self-reported residential histories. Information on plant operations and chemical releases was combined with environmental characteristics of the region through a series of linked models to estimate air and water concentrations of PFOA from 1951 to 2008 (Shin et al. 2011a). Based on estimates of individual air and water intake rates and linkage of residential geocodes for participant address histories to public water distribution systems and private wells, yearly PFOA serum concentrations were estimated for each participant in the C8 Health Project (Shin et al. 2011b). Historical individual modeled serum PFOA was calibrated by factors derived from comparisons of observed with predicted serum concentrations in 2005–2006. The ratio of observed to predicted (before calibration) estimates for these mothers showed a geometric mean (GM) of 1.36, and the interquartile range (IQR) of these ratios was 0.7 to 2.2. *In utero*, modeled exposures for each child were estimated as the modeled serum concentrations in the mother who had been successfully matched to the child, at the time of the first trimester of pregnancy (*n* = 4,713); these pregnancies occurred from 1987 to 2005.

*Thyroid hormone determination and subclinical hypo- and hyperthyroidism.* We assessed thyroid function by measuring thyroid-stimulating hormone (TSH) and total T_4_ (TT_4_), in serum samples (LabCorp, Inc., Burlington, NC, USA). TSH was measured using an electrochemiluminescence immunoassay (ECLIA; Roche Diagnostics, Indianapolis, IN, USA) with an LOD of 0.005 μIU/mL. TT_4_ was measured using a cloned enzyme donor immunoassay (CEDIA; Roche Diagnostics) with an LOD of 0.5 µg/dL. Normal ranges for TSH according to the laboratory were 0.7–5.97, 0.6–4.84, and 0.45–4.5 μIU/mL for children 1–5, 6–10, > 10 years of age, respectively. Normal range for TT_4_ was 4.5–12 µg/dL for all ages. The reproducibility for the analytical methods of TSH (*n* = 60) and TT_4_ (*n* = 21) was 7.2% and 9.2%, for the lowest concentrations (0.035 μIU/mL and 3.7 µg/dL, respectively).

We generated categories of subclinical hypothyroidism and hyperthyroidism based on the measured thyroid hormone levels, after excluding participants who reported any thyroid disease and/or medication. Subclinical hypo/hyperthyroidism cutoffs are preferably based on normal laboratory reference ranges of TSH and FT_4_, but in the absence of FT_4_ we used TSH and TT_4_, as reported previously (Wu et al. 2006). Based on hormone levels, we classified children in the category of subclinical hypothyroidism if the TSH value was above the upper bound of the normal reference range given for the laboratory (which varies according to age groups, i.e., TSH > 5.97, > 4.84, > 4.5 μIU/mL in children < 6, 6–10, > 10 years of age, respectively), whereas TT_4_ was within its reference range (4.5–12 µg/dL). The number of children with high TSH (> 10 μIU/mL) and low TT_4_ (< 4.5 µg/dL) was too small (*n* = 8) for separate analyses, and these children were included in the category of subclinical hypothyroidism. We classified children in the subclinical hyperthyroidism category if the TSH value was below the lower bound of the normal reference range given by the laboratory (which varies according to age groups, i.e., TSH < 0.7, < 0.6, < 0.45 μIU/mL in children < 6, 6–10, > 10 years of age, respectively) and TT_4_ within the normal reference range (4.5–12 µg/dL). Children with low TSH (< 0.1 μIU/mL) and high TT_4_ (> 12 µg/dL) (*n* = 4) were included in the subclinical hyperthyroidism category.

*Self-reported thyroid disease and medication.* Parents or legal guardians completed a questionnaire, including information on diagnoses for thyroid disease. Respondents were asked whether they had ever been told by a health-care provider that the child had thyroid disease. If the answer was yes, they were asked to select between one of these types of thyroid diseases: goiter, Hashimoto’s thyroiditis, Graves disease, or others. In the last category, they were asked to provide the type of thyroid disease (most of whom noted hypothyroidism). We considered three classifications of thyroid diseases: *a*) reported diagnosis with any thyroid disease; *b*) reported Hashimoto’s thyroiditis or hypothyroidism; and *c*) a narrower self-reported thyroid disease definition formed by combining report of any type of thyroid disease diagnosis with reported current use of one of the following medications commonly used to treat thyroid disease: Armour thyroid, Levothroid, Levothyroxine, Levoxil, Methimazole, or Synthroid.

*Covariates.* Covariates available for analysis included age (years), sex, race/ethnicity (non-Hispanic white vs. others), body mass index (BMI) expressed as kilograms per meter squared and transformed to a *z*-score based on the 2000 U.S. Centers for Disease Control and Prevention (CDC) growth charts of BMI-for-age (CDC EpiInfo 2010), month of sampling, average household family income (≤ $10,000, $10,001–20,000, $20,001–30,000, $30,001–40,000, $40,001–50,000, $50,001–60,000, $60,001–70,000, > $70,000, or not known), ever smoking (yes or no), and ever alcohol intake (yes or no).

In models of *in utero* exposure, for a subsample, we also had information on newborn’s birth weight (grams) and gestational age (weeks) and maternal weight gain (pounds), smoking habit (yes or no), and alcohol consumption (yes or no) during pregnancy.

*Statistical analyses.* We conducted a regression analysis among participants 1–17 years of age at survey to assess the relationship between thyroid function and modeled *in utero* PFOA concentrations or measured serum PFOA/PFOS/PFNA concentrations in samples collected during 2005–2006. Levels of TSH and PFAAs showed a non-normal distribution and were natural log–transformed before inclusion in the models. We used simple Pearson correlations to describe pairwise relationships between thyroid hormones and also between PFAAs.

We ran linear regression analyses after exclusion of individuals with reported thyroid disease and/or thyroid medication, to calculate the regression coefficient (beta) and 95% confidence intervals (CIs) for thyroid hormone levels and PFAA quartiles or ln(PFAA) concentrations (the later stratified by sex and age groups). Adjusted differences in thyroid hormone levels between quartile groups of PFAA exposure were expressed as percentages relative to the lowest exposure quartile, calculated as the complement of the exponentiated regression coefficient {100 × [exp(beta)–1]} for TSH and the ratio of beta to the mean [(beta/mean) × 100], for TT_4_. After fitting models of ln(PFAA) on ln(TSH) or TT_4_, regression coefficients were transformed to represent percent change in TSH or TT_4_ associated with the IQR—the 75th compared with the 25th percentile for each PFAA exposure estimate. IQRs were calculated for each sex/age group. For TSH, this is the exponentiated value of the product of the coefficient for the interquartile difference in ln(PFAA). For non-log-transformed TT_4_, we estimated the absolute change in TT_4_ associated with one IQR of ln(PFAA) as the coefficient times the IQR and expressed this as a percent of the mean TT_4_. We also fit linear regression models including other PFAAs. In addition, we performed a sensitivity analysis including children with untreated thyroid diseases (i.e., without reported thyroid medication use), but because no major differences were found, we did not present these results.

We ran logistic regression models to calculate ORs and 95% CIs for three categories of reported disease (any thyroid disease, hypothyroidism, and thyroid disease plus medication) and for subclinical hypothyroidism or hyperthyroidism (based on measured thyroid hormone levels) in association with IQR shifts in PFAA concentrations. Finally, we assessed modeled *in utero* and measured serum PFOA in the same models.

We adjusted final models by child age and sex (when not stratified by this variable) and month of sampling [because there was a trend in measured PFAA during the collection year as well as seasonal variations in thyroid hormone levels (Maes et al. 1997)]. No other variables considered met our operational definition of confounder because there was < 10% change in the PFAA coefficients when including or excluding them from the final regression models. These included maternal (age, weight gain, smoking habit, and alcohol consumption during pregnancy) and child (birth weight, gestational age, BMI, average household family income, race/ethnicity, and smoking habit and alcohol intake) variables. For variables with missing values (Table 1), the above criterion was applied for the subsample of participants without missing values. We used the statistical software package STATA for all statistical analyses (STATA Statistical Software, release 12; StataCorp, College Station, TX, USA). Where associations are referred to as statistically significant, this implies a *p*-value of < 0.05.

**Table 1:** Study population (n =10,725), Mid-Ohio Valley, 2005–2006.

Variable	Value
Children		
Sex		
Boys		5,526 (51.5)
Girls		5,199 (48.5)
Birth weight (g)		3,408 ± 523
Gestational age (weeks)		38.9 ± 1.8
Age (years)		11.4 ± 4.1
Race/ethnicity (white)		10,365 (97.4)
BMI (kg/m2)		21.4 ± 5.4
Alcohol consumption		542 (6.4)
Smoking habit		82 (0.77)
Thyroid disease		61 (0.57)
Household income		
< $20,000		2,993 (35.4)
$20,000–70,000		4,335 (51.2)
> $70,000		1,137 (13.4)
Mothersa		
Age (years)		26.2 ± 5.4
Weight gain (lbs)		30.2 ± 13.1
Alcohol consumption		19 (1.2)
Smoking habit		418 (18.5)
Values are n (%) or mean ± SD. Missing values were not considered for percentage calculation. The percent of missing values in children’s variables was for birth weight: 51%, gestational age: 58%, BMI: 8.2%, ever alcohol consumption: 21%, and ever smoking: 0.1%, household family income at survey: 21%. The percent of missing values in maternal variables during pregnancy was for weight gain: 60%, alcohol consumption: 68%, smoking habit: 53%. aDuring pregnancy.		

## Results

Table 1 shows the characteristics of the study population. A slight majority of participants were boys (52%), and the mean age in the population was 11.4 years. Most of the population (97.4%) was white, whereas other reported race/ethnicity groups were black (1.2%), Hispanic (0.2%), Asian (0.1%), American Indian (0.2%), and other (0.9%). Of the 10,725 children 1–17 years of age in this study, 61 individuals (0.6%) reported a diagnosis of thyroid disease and 39 of the 61 reported a diagnosis of hypothyroidism (including Hashimoto’s thyroiditis). A total of 53 children who reported thyroid disease were > 10 years old, and 46 out of 61 were girls. Using the stricter definition of reported thyroid disease diagnosis plus use of thyroid medication, 0.4% of children (*n* = 40) were counted as cases.

Table 2 shows the levels of thyroid hormones and PFAAs in children. The distribution of TT_4_ levels was close to normality with mean and median values of 7.5 and 7.4 µg/dL, respectively. TSH mean and median values were 2.1 and 1.8 µIU/mL. TSH was negatively correlated with TT_4_ (*r* = –0.07, *p <* 0.001). Modeled *in utero* PFOA concentration had a median of 12 (IQR = 5.4, 37) ng/mL. At the time of the survey, median measured serum PFOA, PFOS, and PFNA concentrations were 29 (IQR = 13, 68), 20 (IQR = 15, 28), and 1.5 (IQR = 1.2, 2.0) ng/mL, respectively. There was a positive correlation between concentrations of the PFAAs at the time of the survey (PFOA vs. PFOS: *r* = 0.24; PFOA vs. PFNA: *r* = 0.09; PFOS vs. PFNA: *r* = 0.41; *p <* 0.001 in all cases), and between modeled *in utero* and at survey PFOA concentrations (*r* = 0.42, *p* < 0.001).

**Table 2:** TSH, TT4, and PFAA concentrations in children 1–17 years of age, Mid-Ohio Valley, 2005–2006 [median (IQR)].

Variable	All children	Boys	Girls
Measured serum TSH levels (µIU/mL)						
1–5 years		1.93 (1.43, 2.62)		2.02 (1.49, 2.72)		1.83 (1.38, 2.54)
6–10 years		2.08 (1.52, 2.79)		2.08 (1.54, 2.77)		2.07 (1.50, 2.81)
> 10 years		1.71 (1.22, 2.38)		1.78 (1.28, 2.47)		1.62 (1.15, 2.31)
1–17 years		1.83 (1.31, 2.55)		1.89 (1.36, 2.59)		1.76 (1.26, 2.50)
Measured serum TT4 levels (µg/dL)						
1–5 years		7.80 (7.00, 8.70)		7.70 (6.80, 8.60)		8.00 (7.10, 8.80)
6–10 years		7.70 (6.80, 8.60)		7.50 (6.70, 8.40)		7.80 (7.00, 8.70)
> 10 years		7.20 (6.30, 8.20)		7.00 (6.10, 7.90)		7.50 (6.60, 8.50)
1–17 years		7.40 (6.50, 8.40)		7.20 (6.30, 8.10)		7.70 (6.80, 8.60)
Modeled in utero PFOA concentrations (ng/mL)						
1–5 years		23.8 (10.1, 57.2)		25.4 (11.0, 63.2)		20.2 (9.35, 53.7)
6–10 years		14.5 (6.39, 44.9)		15.0 (6.67, 47.4)		14.4 (6.21, 41.0)
> 10 years		9.32 (4.61, 27.7)		8.98 (4.63, 25.6)		9.63 (4.61, 26.6)
1–17 years		11.5 (5.36, 37.2)		11.5 (5.46, 38.7)		11.5 (5.27, 35.4)
Measured serum PFOA concentrations (ng/mL)						
1–5 years		33.8 (16.1, 83.3)		36.4 (17.9, 87.8)		32.0 (13.7, 78.3)
6–10 years		32.2 (14.3, 77.7)		34.6 (15.1, 78.2)		30.1 (13.7, 73.4)
> 10 years		26.9 (12.2, 62.7)		30.5 (13.6, 72.1)		23.6 (11.1, 52.3)
1–17 years		29.3 (13.1, 67.7)		32.2 (14.2, 74.8)		26.3 (12.1, 60.5)
Measured serum PFOS concentrations (ng/mL)						
1–5 years		16.3 (11.3, 24.2)		16.7 (11.4, 24.4)		16.0 (11.2, 23.9)
6–10 years		21.8 (16.0, 30.9)		22.9 (16.8, 32.1)		20.7 (15.2, 29.3)
> 10 years		19.6 (14.4, 27.0)		20.6 (15.4, 28.3)		18.5 (13.4, 25.3)
1–17 years		20.0 (14.5, 27.8)		20.8 (15.3, 29.0)		18.9 (13.7, 26.3)
Measured serum PFNA concentrations (ng/mL)						
1–5 years		1.40 (1.10, 1.90)		1.50 (1.10, 1.90)		1.40 (1.10, 1.90)
6–10 years		1.80 (1.30, 2.30)		1.70 (1.40, 2.30)		1.70 (1.30, 2.40)
> 10 years		1.40 (1.10, 1.90)		1.50 (1.20, 1.90)		1.40 (1.10, 1.70)
1–17 years		1.50 (1.20, 2.00)		1.50 (1.20, 2.00)		1.50 (1.10, 1.90)


Associations between quartiles of PFAAs and ln(TSH) and TT_4_ are shown in Table 3, where the lowest quartile is the reference exposure category. Associations between IQR contrasts in ln(PFAAs) are shown in Table 4. There was little evidence for an association of PFOA with either ln(TSH) or TT_4_ in children 1–17 years of age (Tables 3 and 4). However, an IQR contrast of 10 to 57 ng/mL for modeled *in utero* PFOA was associated with a 2% increase in TT_4_ in children up to 5 years of age (95% CI: 0.1, 3.9), with similar but less precise estimates for boys and girls separately in this age group. A change in measured serum PFOA from 16 to 83 ng/mL was associated with a 4% drop in TSH in all children ≤ 5 years, but the association appeared to be limited to girls (Table 4). In children ≤ 5 years, associations between measured serum PFOA and TSH remained significant after adjusting for PFOS (–5.7% change; 95% CI: –9.8, –1.4) and PFNA (–4.7% change; 95% CI: –8.9, –0.4).

**Table 3:** Change in thyroid hormone levels by PFAA quartiles in children 1–17 years of age, Mid-Ohio Valley, 2005–2006.

Children [percent change (95%CI)]		
PFAAs (ng/mL)	TSH (μIU/mL)	TT4 (μg/dL)
Modeled in utero PFOA concentrations				
Q1: 0.05–5.4		Reference		
Q2: 5.5–11.6		0.9 (–3.3, 5.3)		0.1 (–1.4, 1.6)
Q3: 11.7–38.4		–0.2 (–4.5, 4.2)		–0.6 (–2.1, 1.0)
Q4: 38.5–3,987		–1.1 (–5.3, 3.4)		–0.1 (–1.7, 1.4)
Measured serum PFOA concentrations				
Q1: 0.7–13		Reference		
Q2: 13.1–29.2		1.0 (–1.9, 4.0)		0.2 (–0.8, 1.2)
Q3: 29.3–67.6		1.0 (–2.0, 4.1)		0.8 (–0.3, 1.9)
Q4: 67.7–2,071		2.4 (–0.6, 5.5)		0.3 (–0.8, 1.3)
Measured serum PFOS concentrations				
Q1: 0.25–14.4		Reference		
Q2: 14.5–19.9		0.3 (–2.6, 3.2)		0.8 (–0.3, 1.8)
Q3: 20.0–27.7		–1.3 (–4.2, 1.7)		0.9 (–0.2, 1.9)
Q4: 27.8–202		3.1 (0.0, 6.2)		2.3 (1.2, 3.3)
Measured serum PFNA concentrations				
Q1: 0.25–1.1		Reference		
Q2: 1.2–1.4		0.4 (–2.6, 3.5)		0.8 (–0.3, 1.8)
Q3: 1.5–1.9		–0.3 (–3.2, 2.6)		1.7 (0.7, 2.8)
Q4: 2.0–39.8		1.5 (–1.6, 4.6)		2.7 (1.7, 3.8)
TSH was natural log-transformed. Adjusted differences in thyroid hormone levels between quartile (Q) groups of PFAA exposure were expressed as percentages relative to the lowest exposure quartile, calculated from the exponentiated regression coefficient for TSH and from the ratio of beta to the mean for TT4.				

**Table 4:** Change in thyroid hormone levels associated with IQR shifts in PFAAs in children 1–17 years of age, Mid-Ohio Valley, 2005–2006 [percent change (95%CI)].

All childrenb			Boysc			Girlsc		
PFAA	na	TSH (μIU/mL)	TT4 (μg/dL)	TSH (μIU/mL)	TT4 (μg/dL)	TSH (μIU/mL)	TT4 (μg/dL)
Modeled in utero PFOA concentrations														
1–5 years		523		–3.4 (–8.8, 2.4)		2.0 (0.1, 3.9)		–1.0 (–9.0, 7.6)		1.7 (–1.2, 4.6)		–5.8 (–13.4, 2.5)		2.1 (–0.5, 4.8)
6–10 years		1,432		–1.5 (–4.9, 2.1)		0.9 (–0.3, 2.1)		–2.6 (–7.4, 2.4)		0.5 (–1.3, 2.3)		0.2 (–5.1, 4.9)		1.3 (–0.4, 3.0)
> 10 years		2,758		0.1 (–2.2, 2.5)		–0.7 (–1.5, 0.2)		0.2 (–2.8, 3.3)		–0.7 (–1.8, 0.4)		–0.1 (–3.7, 3.7)		–0.8 (–2.2, 0.5)
1–17 years		4,713		–0.5 (–2.4, 1.5)		–0.1 (–0.8, 0.6)		–0.3 (–3.0, 2.5)		–0.3 (–1.3, 0.7)		–0.5 (–3.4, 2.5)		–0.1 (–1.1, 1.0)
Measured serum PFOA concentrations														
1–5 years		1,078		–4.3 (–8.2, –0.3)		0.7 (–0.7, 2.1)		–1.1 (–6.6, 4.7)		1.3 (–0.7, 3.3)		–7.7 (–13.2, –1.7)		–0.1 (–2.2, 2.0)
6–10 years		3,132		0.5 (–2.0, 3.1)		0.9 (0.0, 1.8)		–1.1 (–4.4, 2.3)		0.1 (–1.1, 1.3)		2.2 (–1.7, 6.3)		1.9 (0.6, 3.2)
> 10 years		6,447		2.0 (–0.1, 4.1)		–0.3 (–1.1, 0.4)		1.6 (–1.1, 4.3)		0.5 (–0.5, 1.4)		2.4 (–0.7, 5.7)		–0.9 (–2.0, 0.2)
1–17 years		10,657		1.0 (–0.5, 2.7)		0.1 (–0.5, 0.6)		0.7 (–1.3, 2.7)		0.4 (–0.3, 1.1)		1.3 (–1.0, 3.8)		0.0 (–0.8, 0.7)
Measured serum PFOS concentrations														
1–5 years		1,078		3.1 (–0.9, 7.3)		0.8 (–0.6, 2.2)		1.4 (–4.3, 7.5)		0.4 (–1.7, 2.5)		4.7 (–0.9, 10.5)		1.2 (–0.6, 3.0)
6–10 years		3,132		0.0 (–2.2, 2.3)		0.9 (0.2, 1.7)		–1.7 (–4.5, 1.2)		0.4 (–0.7, 1.4)		2.1 (–1.7, 5.7)		1.5 (0.4, 2.7)
> 10 years		6,447		0.9 (–0.8, 2.7)		1.2 (0.6, 1.9)		1.1 (–1.0, 3.2)		1.2 (0.5, 2.0)		0.8 (–1.9, 3.5)		1.1 (0.1, 2.0)
1–17 years		10,657		1.0 (–0.3, 2.3)		1.1 (0.6, 1.5)		0.4 (–1.2, 2.1)		0.9 (0.3, 1.5)		1.6 (–0.5, 3.6)		1.2 (0.5, 1.9)
Measured serum PFNA concentrations														
1–5 years		1,078		0.2 (–3.5, 4.1)		1.1 (–0.2, 2.4)		–0.7 (–6.0, 4.8)		1.1 (–0.8, 3.0)		1.5 (–3.8, 7.1)		1.1 (–0.7, 2.9)
6–10 years		3,132		0.0 (–2.1, 2.1)		1.0 (0.3, 1.7)		–0.9 (–3.3, 1.6)		0.5 (–0.4, 1.4)		1.0 (–2.4, 4.4)		1.4 (0.3, 2.5)
> 10 years		6,447		1.1 (–0.5, 2.8)		1.3 (0.7, 1.9)		2.0 (0.0, 4.0)		1.9 (1.2, 2.6)		0.2 (–2.0, 2.4)		0.5 (–0.3, 1.3)
1–17 years		10,657		0.8 (–0.4, 2.0)		1.1 (0.7, 1.5)		1.0 (–0.7, 2.6)		1.4 (0.9, 2.0)		0.5 (–1.4, 2.5)		0.9 (0.2, 1.5)
PFAAs and TSH were natural log-transformed. Change in TSH calculated from the exponentiated regression coefficient and change in TT4 as a percent of the mean. aNumber of children with PFAA measurements after excluding those who reported thyroid disease and/or thyroid medication (also excluded from models): boys, n = 5,526; girls, n = 5,199. Models adjusted by sexb, ageb,c, and month of samplingb,c.														

Associations between PFOS or PFNA and TT_4_ were found in children 1–17 years of age (Tables 3 and 4). Interquartile contrasts of 15 to 28 ng/mL in PFOS and 1.2 to 2.0 ng/mL in PFNA were both associated with a 1.1% increase in TT_4_ (95% CIs: 0.6, 1.5 and 0.7, 1.5, respectively). The association was evident in both girls and boys 10–17 years of age in the case of PFOS and for PFNA associations overall were significant for both boys and girls (Table 4). In addition, associations between PFOS or PFNA and TT_4_ were similar after adjustment by other PFAAs percent change for PFOS adjusted by PFNA: 0.7, 95% CI: 0.2, 1.2; or PFOA: 1.1, 95% CI: 0.6, 1.6; percent change for PFNA adjusted by PFOS: 0.8, 95% CI: 0.4, 1.3; or PFOA: 1.1, 95% CI: 0.6, 1.5). We did not find evidence of associations between serum PFOS or PFNA and TSH.

The OR for thyroid disease (*n* = 61) was 1.44 (95% CI: 1.02, 2.03) (Table 5). Most of the children with thyroid disease were reported to have hypothyroidism (*n* = 39; OR = 1.54; 95% CI: 1.00, 2.37). The association was similar for the strictest definition of reported thyroid disease plus use of thyroid medication (OR = 1.61; 95% CI: 1.07, 2.51). Associations were similar for modeled estimates of *in utero* PFOA concentrations, although CIs were a little wider (Table 5). We also performed analyses including both modeled *in utero* and measured PFOA in the same model, and in both cases ORs were attenuated and CIs were wider (OR = 1.29; 95% CI: 0.87, 1.92; and OR = 1.27; 95% CI: 0.74, 2.19 for thyroid disease vs. PFOA modeled *in utero* or at survey, respectively). We did not find associations between concentrations of any of the PFAAs and subclinical hypo/hyperthyroidism (Table 5).

**Table 5:** ORs (95% CIs) of thyroid disease for IQR shift in PFAA concentrations in children 1–17 years of age, Mid-Ohio Valley, 2005–2006.

Reported							Subclinical						
Thyroid disease			Hypothyroidism			Hypothyroidisma			Hyperthyroidisma		
PFAA	n	OR (95% CI)	n	OR (95% CI)	n	OR (95% CI)	n	OR (95% CI)
Modeled in utero PFOA		27		1.47 (0.95, 2.27)		20		1.61 (0.96, 2. 63)		155		0.94 (0.76, 1.16)		31		1.10 (0. 69, 1.74)
Measured serum PFOA		61		1.44 (1.02, 2.03)		39		1.54 (1.00, 2.37)		365		0.98 (0.86, 1.15)		78		0.81 (0.58, 1.15)
Measured serum PFOS		61		0.8 (0.62, 1.08)		39		0.91 (0.63, 1,31)		365		0.99 (0.86, 1.13)		78		0.80 (0.62, 1.02)
Measured serum PFNA		61		1.05 (0.78, 1.41)		39		1.11 (0.77, 1.60)		365		0.99 (0.88, 1.12)		78		0.78 (0.61, 1.01)
PFAAs were natural log-transformed. Models were adjusted by age and sex. aBased on hormonal levels at survey after excluding people who self-reported thyroid disease and/or thyroid medication.																

## Discussion

To the best of our knowledge, this is the first large-scale report of an association between PFAAs in serum and thyroid function impairment in children 1–17 years of age. In a group of 10,725 children from the Mid-Ohio Valley, measured serum PFOA concentrations in 2005–2006 were positively associated with thyroid disease (mostly hypothyroidism). In addition, serum concentrations of PFOS and PFNA, but not PFOA, were positively associated with TT_4_ levels in children 1–17 years of age.

Serum PFOS and PFNA concentrations were associated with slightly higher levels of TT_4_ in children 1–17 years of age, but were not positively associated with subclinical hyperthyroidism. PFOA was not associated with TSH or TT_4_ in all children combined, though subgroup analyses suggested possible associations with PFOA measured at survey and modeled *in utero,* in children ≤ 5 years. However, given the lack of effect in the other age groups, this may be a chance finding. Previous literature on child thyroid function and PFAA exposure is limited. A recent study in Korea (*n* = 29) reported a positive association between maternal serum PFOA (median = 1.46 ng/mL) concentrations and cord serum TSH levels. No association was found between PFOA, PFOS, or PFNA and T_4_ (Kim et al. 2011). Another small study of 15 Japanese mother–child pairs reported no apparent correlation between maternal blood PFOS (median = 8.1 ng/mL) concentrations and neonatal blood TSH or FT_4_ (Inoue et al. 2004). In agreement with our results, a recently published study of 52,296 adults from the C8 Health Project found a positive association between serum PFOS and TT_4_. They also found a positive association between serum PFOA and TT_4_ in women of all ages, and men > 50 years of age (Knox et al. 2011). In addition, a positive association was reported between plasma PFOS (GM = 18.3 ng/mL) and FT_4_ levels and inverse with TSH in environmentally exposed adults (*n* = 621) from Nunavik, Quebec, Canada (Dallaire et al. 2009). On the contrary, a modest inverse association between serum PFOA (median = 1.1 µg/mL) and FT_4_ but not TT_4_ or TSH levels was reported in an occupational study among 506 employees in one Belgian and two American factories (Olsen and Zobel 2007). Some adult studies have not found associations between PFAA exposures and thyroid level alteration, including a study of Canadian pregnant women with hypothyroxinemia (*n* = 96; PFOA and PFOS medians = 3.9 and 15.5 nmol/L) whose serum PFAA concentrations were comparable to matched controls (*n* = 175; PFOA and PFOS medians = 3.6 and 16.4 nmol/L) (Chan et al. 2011). There was no evidence of an association between elevated serum PFOA (median = 354 ng/mL) and TSH levels in residents (*n* = 371) from the same area as the present study (Emmett et al. 2006). In a small study (*n* = 31) of anglers from New York State, no association was reported between PFOA, PFOS, or PFNA concentrations (GM = 1.3, 19.6, and 0.79 ng/mL) and serum TSH or FT_4_ levels (Bloom et al. 2010).

Altered thyroid hormone levels following exposure to PFAAs have also been found in experimental animal studies where pre- and postnatal long-term exposure to PFOS decreased serum levels of TT_4_ and FT_4_ in pregnant dams and pups, without a concomitant rise in TSH (Lau et al. 2007). PFNA exposure did not alter TT_4_ levels in zebrafish juveniles treated with PFNA until maturity (Liu et al. 2011). In adult monkeys, PFOA exposure during 6 months, which led to serum levels up to 158 µg/mL, did not alter TT_4_ or TSH (Butenhoff et al. 2002).

We found higher odds of reporting thyroid disease (mostly hypothyroidism) with increased measured PFOA concentrations, even when we restricted cases to children with reported disease plus use of thyroid medication. However, PFOA concentrations were not associated with subclinical hypo/hyperthyroidism based on individual hormone levels or thyroid hormone levels (as continuous variables) in children 1–17 years of age. Therefore, the association between reported thyroid disease and PFOA exposure should be considered with caution. Nevertheless, these results are comparable to a cross-sectional analysis of PFOA/PFOS concentrations and reported thyroid disease in adults in NHANES for 1999–2000, 2003–2004, and 2005–2006 (*n* = 3,966). An OR of 2.2 (95% CI: 1.4, 3.7) was estimated for thyroid disease in association with the highest versus first and second quartiles of serum PFOA in females (mean = 3.77 ng/mL), and a similar association with serum PFOS (mean = 25.1 ng/mL) was reported for males (OR = 2.7; 95% CI: 1.0, 7.0) (Melzer et al. 2010).

The main strengths of the present study are the large sample size and the data for both measured and modeled *in utero* PFOA concentrations. A further strength is the high rate of participation, diminishing concern about potential selection biases. It is believed that this population is representative, given the high participation rates in the C8 Health Project, of all those children who drank contaminated water in the Mid-Ohio Valley. Moreover, we were able to adjust for a number of potential confounders.

The mostly cross-sectional design of the present study is a major limitation because the single measurements preclude determination of the time sequence between PFAA exposure and outcome, and some associations may have been attributable to chance or uncontrolled sources of bias. One further limitation is the absence of measurements of child triiodothyronine and FT_4_ levels, which would have yielded more comprehensive information concerning the child’s thyroid regulatory system. Another limitation is the reliance on recall for thyroid diagnosis, although we also investigated a more stringent case definition of report plus medication use and obtained similar results. However, the prevalence of hypothyroidism reported in our study population (0.4%) was higher than the range (0.04–0.14%) of the estimated prevalence in several children and adolescent populations (Hunter et al. 2000). Based on TSH and TT_4_ levels, the prevalence of subclinical hypothyroidism in the 1988–1994 NHANES population (Wu et al. 2006) was lower than that of the present study (1.7% vs. 2.9%, respectively, in children > 12 years of age), whereas subclinical hyperthyroidism was more prevalent (2.3% vs. 1.2% in children > 12 years).

## Conclusions

In summary, this is the first large-scale report on thyroid hormone function and PFAA concentrations among children 1–17 years of age. Results suggest that serum PFOS and PFNA concentrations are associated with thyroid hormone levels, and serum PFOA concentrations are associated with reported hypothyroidism. However, further studies to understand the effect of pre- or postnatal exposure to PFAAs on thyroid hormones in children are warranted.
